# A Practical Manual of Insanity

**Published:** 1902-07

**Authors:** 


A Practical Manual of Insanity. For the Student and General Practitioner. By Daniel R. Brower, A. M., M. D., LL. D., Professor of Nervous and Mental Diseases in Rush Medical College, in Affiliation with the University of Chicago, and in the Post-Graduate Medical School, Chicago; and Henry M. Bannister, A. M., M. D., formerly Senior Assistant Physician, Illinois Eastern Hospital for the Insane. Octavo, 426 pages, with a large number of full-page inserts. Philadelphia and London: W. B. Saunders & Company. 1902. [Cloth, $3 00 net.]

This work is essentially clinical in its presentation of the subject. The various forms of insanity are clearly described and a simple classification has been adopted. The authors have included a chapter giving the classification of different writers in a very useful table of parallel columns. There is an excellent chapter on the general diagnosis and prognosis of mental disease.

General rules of procedure in the examining of supposedly insane persons are very difficult to formulate. The advice, "It is not always necessary to state to him that he is suspected of insanity, but it is seldom, if ever, advisable to use deception or appear before him in a false character," is sound and is a rule that should be followed. We believe that the insane must be treated in a frank manner; that they must be told, except in cases of acute mania or in those forms of insanity in which there is no understanding, that the object of the visit is to ascertain the general health of the patients. The rules for managing the patients who are sent to institutions and the general care of patients when home treatment has been decided upon, are practical and helpful.

The discussion of the subject of paranoia is scientific and shows that the experience of the authors with this type has been large and their observations accurate. The book closes with a chapter on the ethics of insanity. The question of marriage of those with an ancestral taint of insanity is discussed. The question as to the continuance of sexual relations of persons who have recovered from mental disease is considered. The authors reply cautiously that in some cases it may be safe.

The ethical question of secrecy is an interesting one, and the obligation of the physician is clearly stated. The volume is practical in character and covers the general subject most thoroughly. J.W.P.

